# Association of Caveolin-1 Expression With Prostate Cancer: A Systematic Review and Meta-Analysis

**DOI:** 10.3389/fonc.2020.562774

**Published:** 2021-01-08

**Authors:** Pei Chen, Yu-ling Zhang, Bai Xue, Guo-ying Xu

**Affiliations:** ^1^ Department of Basic Medicine, Jiangsu College of Nursing, Huai’an, China; ^2^ Department of Medical Technology, Jiangsu College of Nursing, Huai’an, China

**Keywords:** prostate cancer, caveolin-1, prognosis, meta-analysis, clinicopathological parameters

## Abstract

**Purpose:**

The prognostic value of caveolin-1 in prostate cancer remains uncertain. Hence, this meta-analysis was performed to evaluate the prognostic value of caveolin-1 in prostate cancer, as well as ascertain the relationship between caveolin-1 expression and clinicopathological characteristics of prostate cancer patients.

**Methods:**

The PubMed, Embase, Chinese National Knowledge Infrastructure and Chinese Biology Medicine databases were electronically searched to retrieve published studies on caveolin-1 expression in prostate cancer. After study selection and data extraction, the meta-analysis was conducted using Review manager 5.3 software. Odds ratio (OR) with 95% confidence interval (CI) was used to estimate the pooled effect. Funnel plot was used to assess publication bias.

**Results:**

A total of ten studies were enrolled, which included 3976 cases of prostate cancer, 72 cases of high-grade intraepithelial neoplasia (HGPIN), and 157 normal controls. Results of the meta-analysis showed that the positive rate of caveolin-1 expression in prostate cancer was 18.28 times higher than that in normal control (OR= 18.28, 95% CI: 9.02–37.04, p<0.01), and 4.73 times higher than that in HGPIN (OR= 4.73, 95% CI: 2.38–9.42, p<0.01). The relationship between caveolin-1 and clinicopathological characteristics of prostate cancer showed that the differences in caveolin-1 expression in patients with prostate-specific antigen (PSA) >10 vs. ≤ 10 (OR=2.09, 95% CI: 1.35–3.22, p<0.01), differentiation degree low vs. medium/high (OR=2.74, 95% CI: 1.84–4.08, p<0.01), TNM stage T3+T4 vs. T1+T2 (OR=2.77, 95% CI: 1.78–4.29, p<0.01), and lymph node metastasis present vs. absent (OR=2.61, 95% CI: 1.84–3.69, p<0.01) were statistically significant. The correlation analysis between caveolin-1 and the survival time of patients with prostate cancer demonstrated that caveolin-1 was closely related to the prognosis of prostate cancer patients (HR=1.50, 95% CI: 1.28–1.76, p<0.01).

**Conclusion:**

Caveolin-1 is overexpressed in prostate cancer, which can serve as a risk factor and adverse clinicopathological feature of prostate cancer. Caveolin-1 can also predict poor survival in prostate cancer patients after radical prostatectomy.

## Introduction

According to the official cancer epidemic statistics, about 914,000 new cases of prostate cancer were detected in 2008, accounting for 13.8% of all male cancer incidence, and the fifth highest of all cancer incidence (regardless of gender) (1). The majority (80%) of newly diagnosed cases are localized prostate cancer, while the others are advanced or metastatic prostate cancer (2). Overall survival (OS) rate is very high in localized disease, but decreases dramatically in advanced and metastatic cases, with the 5-years survival rate of 26%–30% (3). Therefore, it is critical to explore effective biomarkers for early diagnosis, and for predicting tumor progression and prognosis, which will significantly reduce mortality, especially in advanced or metastatic prostate cancer patients.

There are three known caveolins (caveolin-1, 2, and 3) that are ubiquitously expressed in all cell types (4, 5), and are involved in binding, localizing and regulating various signaling proteins (6). Caveolin-1 is an integral membrane protein encoded by the CAV1 gene, which is related to the formation of caveolae. The caveolin-1 gene is located at chromosome 7q31.1 and has two subtypes (a and b), which have a high incidence of tumor suppressor gene loss in various tumor types. The structure and function of the caveolin-1 gene family are highly conserved in different species, indicating that it is essential for maintaining cellular functions. Caveolin-1 mainly interacts with various signal transduction molecules through phosphorylation/dephosphorylation signaling. It plays an important role in cholesterol and lipid transport, membrane transport, signal transduction, and cell adhesion. Moreover, caveolin-1 is involved in the regulation of various signaling pathways such as cell proliferation, differentiation, apoptosis, migration, and angiogenesis, and is closely correlated with the occurrence, development, invasion, and metastasis of tumors. The carcinogenic role of caveolin-1 has been identified in many tumors, suggesting that it may act as a novel therapeutic target for tumors.

Caveolin-1 is reportedly expressed in prostate cancer, and may be related to the clinicopathological characteristics of prostate cancer patients. However, due to sample size limitation, ethnic differences and other factors, the results from different studies have been inconsistent. Hence, this meta-analysis was conducted to comprehensively evaluate the role of caveolin-1 in early diagnosis, progression prediction, and prognosis of prostate cancer, as well as to clarify the relationship between caveolin-1 and the clinicopathological characteristics of prostate cancer patients.

## Materials and Methods

This meta-analysis was based on the Preferred Reporting Items for Systematic Reviews and meta-analysis (PRISMA) guidelines (7).

### Inclusion and Exclusion Criteria

Inclusion criteria: (1) Patients with prostate cancer confirmed by pathology. (2) Immunohistochemistry was used to detect the expression of caveolin-1. (3) Full texts were available. (4) The relationship between caveolin-1 and clinicopathological significance in prostate cancer was evaluated.

Exclusion criteria: (1) Animal experiment; (2) Comment, review or case report; (3) The effective data could not be extracted.

When duplicate studies were identified, the study with the most complete data and higher methodological quality was included.

### Literature Search

The PubMed, Embase, Chinese National Knowledge Infrastructure and Chinese Biology Medicine databases were electronically searched from their inception to June 2020. The following search strategy and key words were used: (“Prostate Cancer” OR “Cancer, Prostate” OR “Prostate Carcinoma” OR “Carcinoma, Prostate” OR “Prostate Neoplasm” OR “Neoplasm, Prostate”) AND (“Caveolin-1” OR “Caveolin” OR CAV1). The reference lists of retrieved articles were also manually reviewed to identify additional potentially relevant studies.

### Data Extraction

Using a pre-specified, standardized data extraction form, two researchers independently completed the data extraction and sorting. Any disagreements were resolved by discussion to achieve a consensus. The extracted data mainly included the first author’s name, country, date of publication, number of patients and controls, tumor differentiation, serum prostate-specific antigen (PSA) level, clinical stage, and lymph node metastasis. The Newcastle-Ottawa Scale (NOS) scale was used to evaluate the quality of enrolled studies.

### Statistical Analysis

Review manager 5.3 software was used for statistical analysis. Pooled odds ratio (OR) and 95% CI were used to evaluate the expression of caveolin-1 in prostate cancer patients and controls. The χ2 and I^2^ tests were used to judge heterogeneity between studies. If I^2^<50% and p>0.10, there was no statistical heterogeneity, and a fixed effects model was selected for data consolidation. If I^2^≥50% and p≤0.10, there was statistical heterogeneity, and a random effects model was used for data consolidation. If the heterogeneity was very high, only descriptive score was given. The inverted funnel chart was used to analyze publication bias.

## Results

### Characteristics of Enrolled Studies

**Figure 1:**
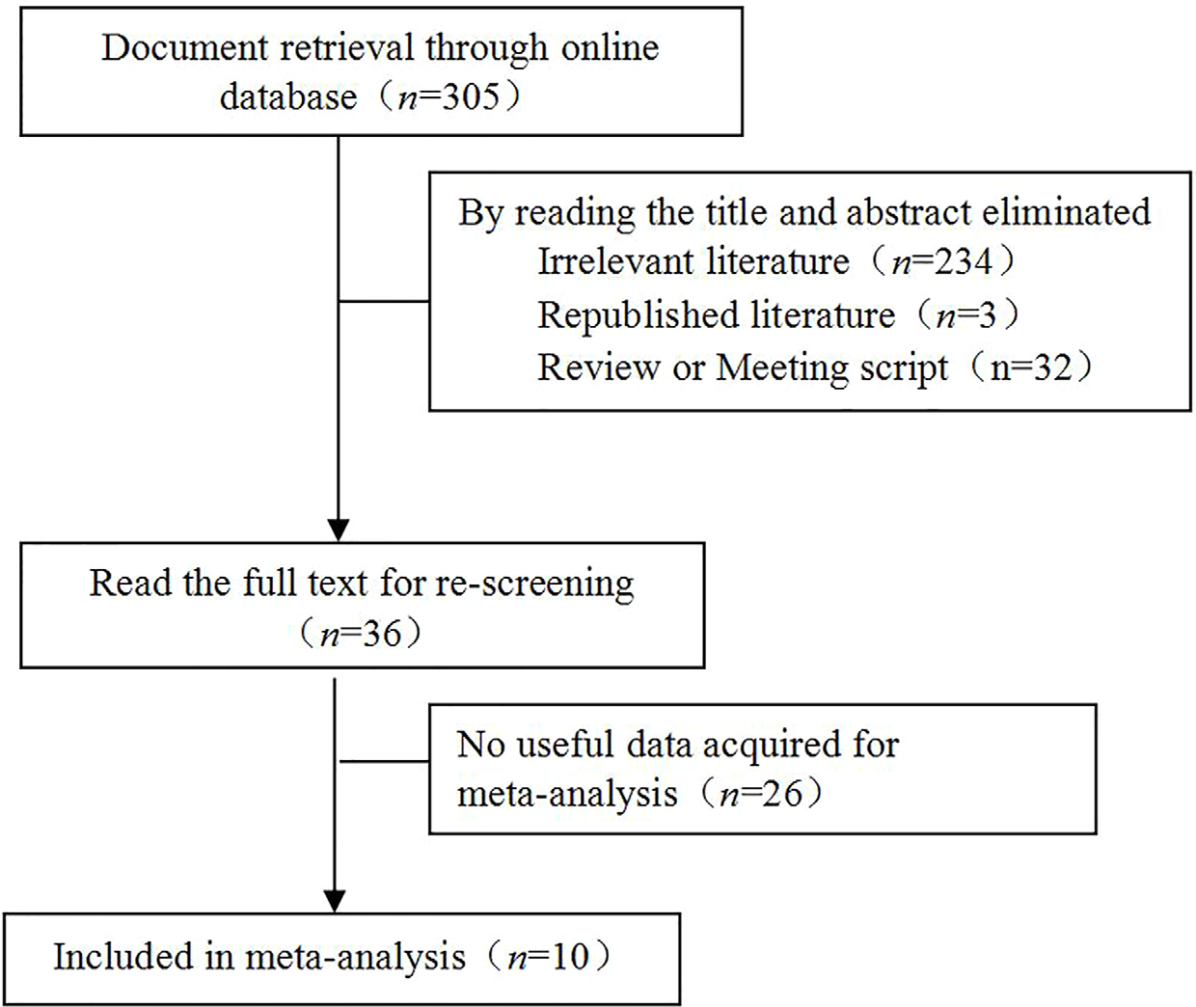
Literature retrieval process. A total of 305 studies were initially retrieved. Through screening and data extraction, ten studies were finally enrolled for the meta-analysis.

Based on the inclusion and exclusion criteria, 10 studies (8–17) were enrolled, which included 3976 cases of prostate cancer, of which 962 were positive for caveolin-1. Moreover, 72 cases were high-grade intraepithelial neoplasia of prostate (HGPIN), of which 15 were positive for caveolin-1. A total of 157 normal controls were included, of which 23 were positive for caveolin-1. Eight studies reported the relationship between the differentiation degree of prostate cancer cells and caveolin-1 expression. Seven studies reported the relationship between serum level of PSA and caveolin-1 expression. Seven studies reported the relationship between TNM clinical stage and caveolin-1 expression. Six studies reported the relationship between lymph node metastasis and caveolin-1 expression. The literature retrieval process is shown in **Figure 1**, and the basic characteristics of the studies are shown in **Table 1**.

**Table 1:** Detailed characteristics of enrolled studies.

First Author	N	Caveolin-1 Positive			Differentiation(High/Low)	PSA0–10/>10(ng/ml)	Clinical stageI–II/III–IV	Lymph node metastases(+/-)	Survival analysis
		Control	Prostate cancer	HGPIN					
Wang XM (8)	67	——	29/47	2/20	27/20	6/41	26/21	——	–
Mohammed DA (9)	65	5/25	18/20	12/20	7/13	10/10	——	16/4	–
Liu ZG (10)	67	2/20	29/47	——	27/20	6/41	26/21	——	–
Zeng YW (11)	143	6/69	41/59	6/15	29/30	5/54	34/25	22/37	+
Wei SP (12)	72	1/30	17/42	——	24/18	11/31	25/17	——	–
Karam JA (13)	232	——	70/232	——	210/20	——	——	6/226	+
Satoh T (14)	152	——	46/152	——	132/20	87/64	137/15	10/139	+
Yang G (15)	189	——	47/189	——	166/23	75/53*	165/17	27/162	+
Yang G (16)	111	1/13	23/71	3/17	——	——	29/42	25/46	–
Mathieu R (17)	3,117	——	642/3,117	——	——	——		73/3,044	+

HGPIN, high-grade intraepithelial neoplasia; PSA, prostate specific antigen.

### Quality Evaluation of Included Studies

The NOS scale was used to evaluate the quality of the included studies, mainly based on the selection of cases and controls, the representativeness of cases, the control of confounding factors, the consistency of detection methods and other aspects. The results are shown in **Table 2**.

**Table 2:** Quality evaluation of included studies.

First Author	①	②	③	④	⑤A	⑤B	⑥	⑦	⑧	NOS Scores
Wang XM (8)	1	1	0	0	1	0	1	0	1	5
Mohammed DA (9)	1	1	1	1	1	0	1	1	1	8
Liu ZG (10)	1	1	1	1	1	0	1	1	1	8
Zeng YW (11)	1	1	1	1	1	0	1	1	1	8
Wei SP (12)	1	1	1	1	1	0	1	1	1	8
Karam JA (13)	1	1	0	0	1	0	1	0	1	5
Satoh T (14)	1	1	0	0	1	0	1	0	1	5
Yang G (15)	1	1	0	0	1	0	1	0	1	5
Yang G (16)	1	1	1	1	1	0	1	1	1	8
Mathieu R (17)	1	1	0	0	1	0	1	0	1	5

(1) Whether the determination of cases was appropriate; (2) Representativeness of cases; (3) Selection of control; (4) Determination of control; (5) In the design and analysis, the comparability of case and control was considered. A: control the most important confounding factors, B: control any confounding factors; (6) Definition of exposure; (7) Whether the same method was used for case and control exposure; (8) No response rate.

### Sensitivity Analysis and Publication Bias

Sensitivity analysis was performed to assess the influence of each study on the overall result by sequentially eliminating each study. The results showed that no individual study significantly affected the pooled ORs, suggesting that the results of this meta-analysis were stable and reliable.

**Figure 2:**
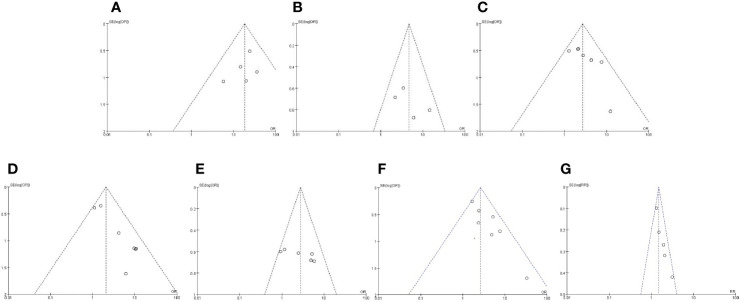
Funnel plot for publication bias detection. Funnel chart was used to evaluate the publication bias of the studies. The shape of the funnel chart did not show any evidence of significant asymmetry in the dominant model. **(A)** Expression of caveolin-1 between prostate cancer and normal control; **(B)** Expression of caveolin-1 between prostate cancer and high-grade intraepithelial neoplasia (HGPIN); **(C)** Expression of caveolin-1 in prostate cancer with differentiation; **(D)** Expression of caveolin-1 in prostate cancer with prostate-specific antigen (PSA); **(E)** Expression of caveolin-1 in prostate cancer with TNM stage; **(F)** Expression of caveolin-1 in prostate cancer with lymph node metastasis. **(G)** Caveolin-1 acts as a predictor for tumor prognosis.

Funnel chart was used to evaluate the publication bias of the studies. The shape of the funnel chart did not show any evidence of significant asymmetry in the dominant model (**Figure 2**).

### Results of Meta-Analysis

#### Comparison of Caveolin-1 Expression Between Prostate Cancer and Control

**Figure 3:**
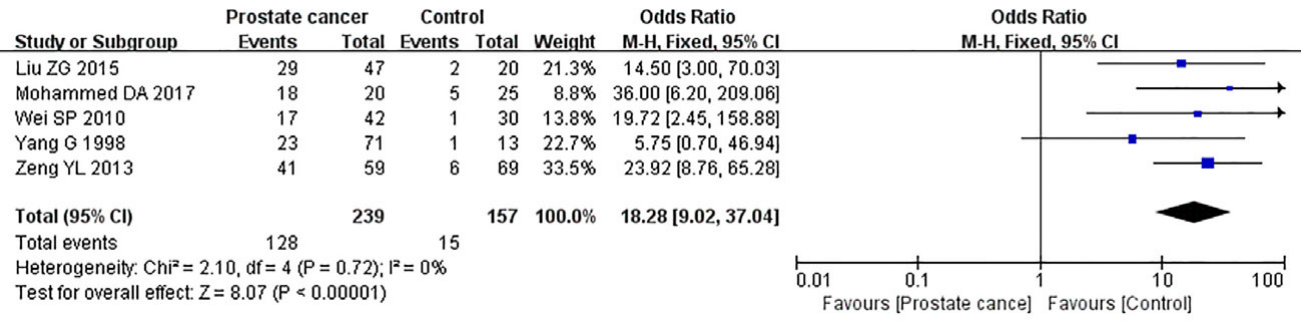
Forest plot on the comparison of caveolin-1 expression between prostate cancer and normal control. Five studies were enrolled to evaluate the expression of caveolin-1 in prostate cancer and control. No heterogeneity was observed among the included studies (I^2^=0%, p=0.72). Results showed that the expression of caveolin-1 in prostate cancer was 18.28 times higher than that in normal control.

Five studies reported the expression of caveolin-1 in prostate cancer cases and controls, which included 239 cases of prostate cancer, of which 128 cases were positive for caveolin-1, with a positive rate of 53.56%. Among the 157 controls, 15 were positive for caveolin-1, with a positive rate of 9.55%. There was no heterogeneity among the included studies (I^2^ = 0%, p=0.72). The fixed effects model was used for meta-analysis. The results showed that the expression of caveolin-1 in prostate cancer was 18.28 times higher than that in normal controls (OR=18.28, 95% CI: 9.02–37.04, p<0.01) (**Figure 3**).

#### Comparison of Caveolin-1 Expression Between Prostate Cancer and HGPIN

**Figure 4:**
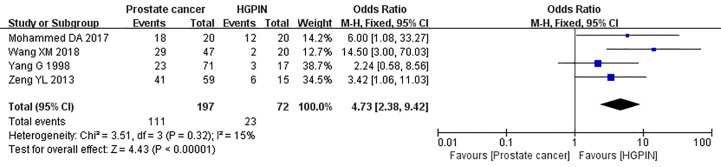
Forest plot on the comparison of caveolin-1 expression between prostate cancer and high-grade intraepithelial neoplasia (HGPIN). Four studies were enrolled to evaluate the expression of caveolin-1 in prostate cancer and HGPIN. No heterogeneity was observed among the include studies (I^2^=15%, p=0.32). Results showed that the expression of caveolin-1 in prostate cancer was 4.73 times higher than that in HGPIN.

Four studies reported the expression of caveolin-1 in prostate cancer and HGPIN, which included 197 cases of prostate cancer, of which 111 cases were positive for caveolin-1, with a positive rate of 56.35%. Among the 72 cases of HGPIN, 23 cases were positive for caveolin-1, with a positive rate of 31.94%. There was no heterogeneity among the included studies (I^2^ = 15%, p=0.32). The fixed effects model was used for meta-analysis. The results showed that the expression of caveolin-1 in prostate cancer was 4.73 times higher than that in HGPIN (OR=4.73, 95% CI: 2.38–9.42, p<0.01) (**Figure 4**).

### Relationship Between Caveolin-1 Expression and Clinicopathological Characteristics of Prostate Cancer

**Figure 5:**
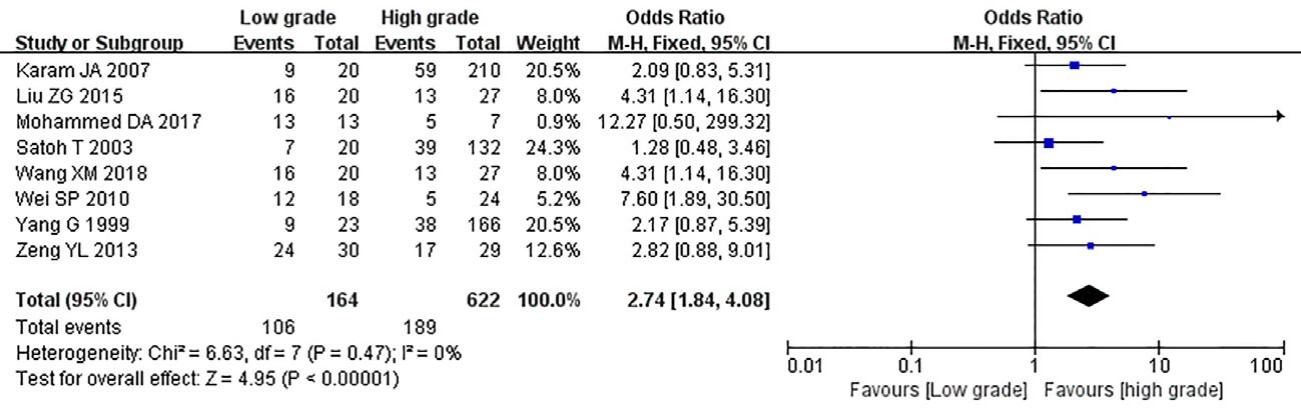
Forest plot on the effect of differentiation on caveolin-1 expression in prostate cancer. Eight studies reported the expression of caveolin-1 in prostate cancer with cancer differentiation. No heterogeneity was observed among the included studies (I^2^= 0%, p=0.44). Results showed that the rate of caveolin-1 expression in low grade differentiated prostate cancer cases was 2.74 times higher than that in high grade.

**Figure 6:**
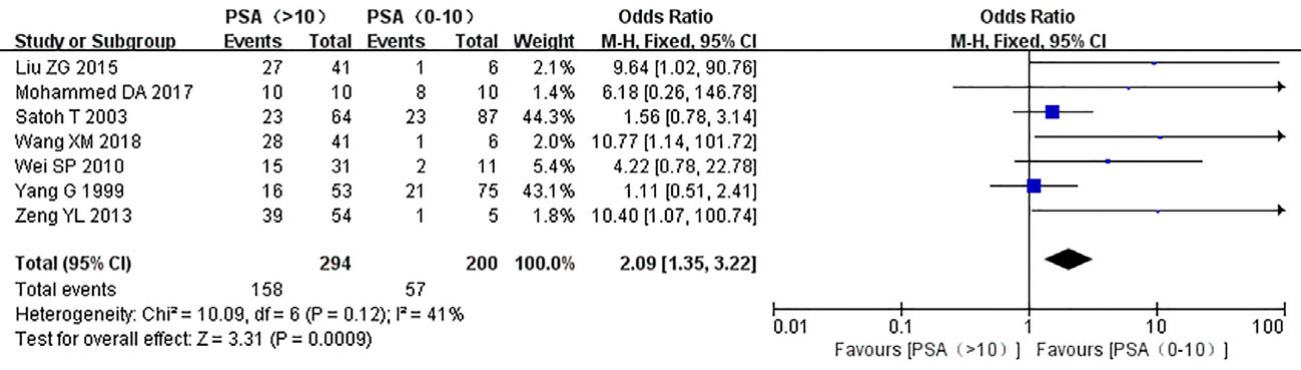
Forest plot on the effect of prostate-specific antigen (PSA) level on caveolin-1 expression in prostate cancer. Seven studies reported the effect of PSA level on the expression of caveolin-1 in prostate cancer. No heterogeneity was observed among the included studies (I^2^=41%, p=0.12). Results showed that the rate of caveolin-1 expression in prostate cancer with PSA >10 ng/ml was 2.09 times higher than that in prostate cancer with PSA ≤10 ng/ml.

**Figure 7:**
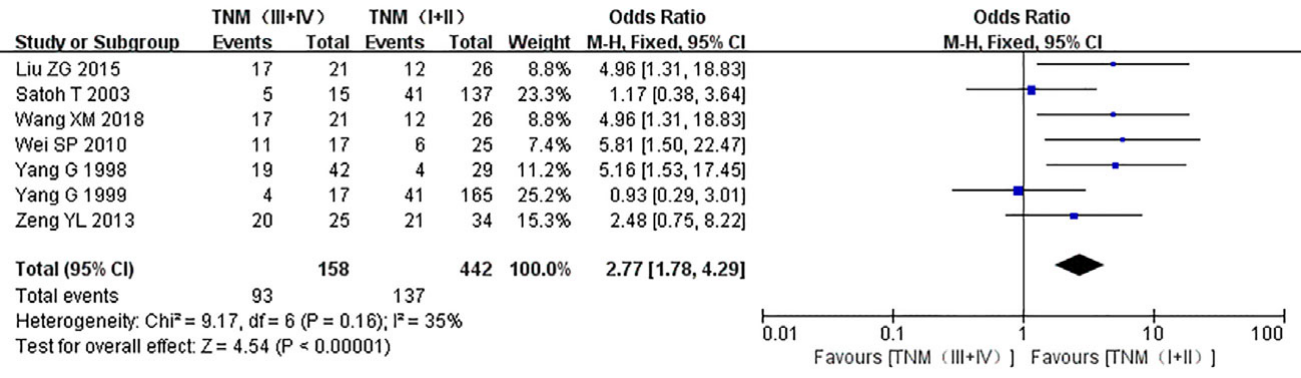
Forest plot on the effect of clinical stage on the expression of caveolin-1 in prostate cancer. Seven studies reported the expression of caveolin-1 in prostate cancer with different TNM stages. No heterogeneity was observed among the included studies (I^2^=35%, p=0.16). Results showed that the rate of caveolin-1 expression was 2.77 times higher in TNM stages (III + IV) than that in TNM stages (I + II).

**Figure 8:**
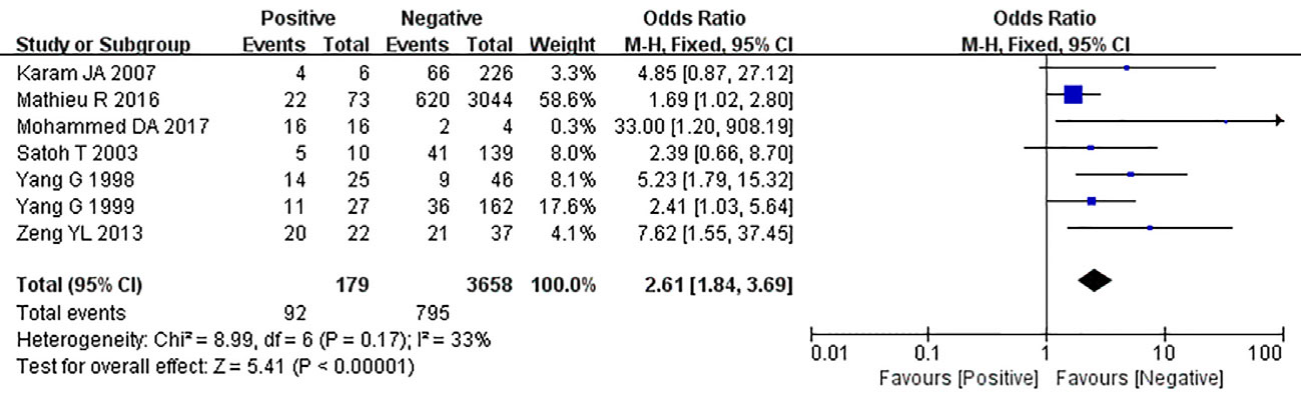
Forest plot on the effect of lymph node metastasis on caveolin-1 expression in prostate cancer. Six studies reported the expression of caveolin-1 in prostate cancer with lymph node metastasis. No heterogeneity was observed among the included studies (I^2^=33%, p=0.17). Results showed that the rate of caveolin-1 expression was 2.61 times higher in positive lymph node metastasis (+) than that in negative lymph node metastasis (-).

The relationship between the expression of caveolin-1 and clinicopathological characteristics of prostate cancer, including tumor differentiation, level of serum PSA, TNM clinical stage, and lymph node metastasis was explored. The results demonstrated that overexpression of caveolin-1 was correlated with low grade differentiation. The rate of caveolin-1 expression in low grade differentiated prostate cancer cases was 2.74 times higher than that in high grade (OR=2.74, 95% CI: 1.84–4.08, p<0.01, **Figure 5**). Overexpression of caveolin-1 was correlated with serum PSA >10 ng/ml. The rate of caveolin-1 expression in prostate cancer with PSA >10 ng/ml was 2.09 times higher than that in prostate cancer with PSA ≤10 ng/ml (OR = 2.09, 95% CI: 1.35–3.22, p< 0.01, **Figure 6**). Overexpression of caveolin-1 was also correlated with TNM stages (III+IV) and lymph node metastasis (+). The rate of caveolin-1 expression was 2.77 and 3.91 times higher in TNM stages (III+IV) and lymph node metastasis (+) than that in TNM stages (I + II) and lymph node metastasis (-) [(OR=2.77, 95% CI: 1.78-4.29, p<0.01, **Figure 7**) and (OR=2.61, 95% CI: 1.84–3.69, p<0.01, **Figure 8**)], respectively. No obvious heterogeneity was observed among the included studies while analyzing the relationship between caveolin-1 and tumor differentiation, level of serum PSA, TNM clinical stage and lymph node metastasis.

### Caveolin-1 Acts as a Predictor for Tumor Prognosis

**Figure 9:**
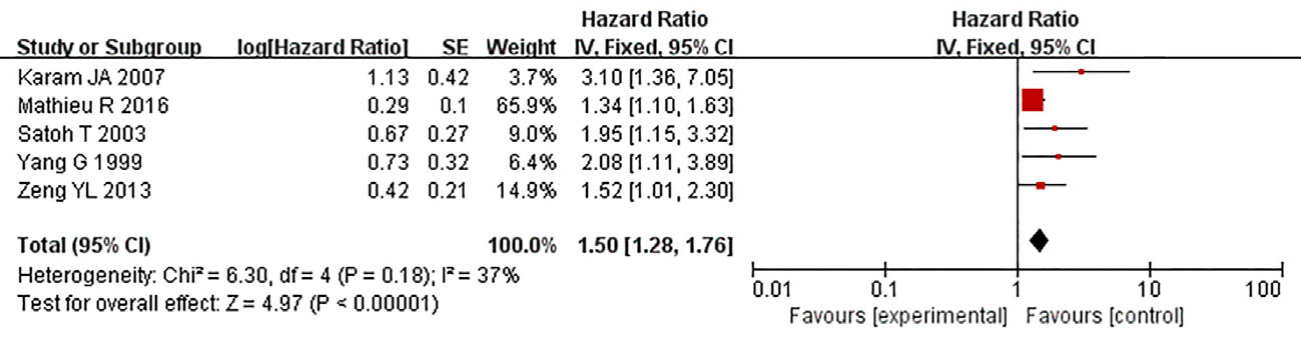
Forest plot on the role of caveolin-1 as a predictor for tumor prognosis. Five studies reported the relationship between the overexpression of caveolin-1 and the survival time of prostate cancer patients after radical prostatectomy. No heterogeneity was observed among the included studies (I^2^ = 37%, p=0.18). Results indicated that after radical prostatectomy, the survival time of patients with caveolin-1 overexpression was significantly lower than those with normal expression.

Five studies reported the relationship between the overexpression of caveolin-1 and the survival time of prostate cancer patients after radical prostatectomy. Meta-analysis showed that HR = 1.50, 95% CI: 1.28–1.76, p<0.01 (**Figure 9**), indicating that the survival time of patients with caveolin-1 overexpression was significantly lower than that of patients with normal expression after radical prostatectomy, suggesting that caveolin-1 is closely related to the prognosis of prostate cancer patients.

## Discussion

Caveolins are a family of scaffolding proteins that coat 50–100 nm plasma membrane invaginations (18). Caveolin-1 is an essential structural component of caveolae. Earlier studies indicated that caveolin-1 was related to endocytosis, signaling and lipid disorders, while recent studies focused on clarifying its relevance in cancer. Caveolin-1 is expressed in esophageal carcinoma (19, 20), prostate cancer (8–17), colon cancer (21), breast cancer (22), bladder cancer (23), lung cancer (24), and other cancers (25, 26), and acts as a tumor promoter or suppressor. As a tumor promoter, high expression of caveolin-1 drives tumorigenesis by inhibiting apoptosis, facilitating anchorage-independent growth, drug resistance as well as metastasis (27, 28). In contrast, caveolin-1 also acts as a tumor suppressor and its low expression favors tumor progression (29, 30).

Prostate cancer is the most common cancer in men, accounting for 10%–20% of all cancer types. Recent studies have shown that caveolin-1 is closely related to the occurrence and development of prostate cancer. The serum level of caveolin-1 in prostate cancer patients is significantly higher than that in normal and benign prostatic hypertrophy (BPH) patients (31). Moreover, the expression of caveolin-1 is positively correlated with prostate cancer progression and metastasis (32). The expression of caveolin-1 in erosive cells was about two times higher than that in non-erosive cells. Caveolin-1 mediates the aggregation of membrane proteins and the signaling of cell proliferation, leading to the occurrence and development of tumors. Therefore, overexpression of caveolin-1 in aggressive prostate cancer cells can confer a survival advantage to these cells, leading to disease progression (33). Due to insufficient samples, ethnic differences, different detection methods, and other factors, previous studies on the relationship between caveolin-1 and prostate cancer showed significantly divergent or conflicting results. Hence, this meta-analysis was conducted to verify the correlation between caveolin-1 and prostate cancer through comprehensive statistical analysis.

The results of this meta-analysis indicated that the expression of caveolin-1 in prostate cancer was 18.28 times higher than that in the normal controls and 4.73 times higher than that in HGPIN, suggesting that caveolin-1 plays an important role in HGPIN and prostate cancer. PSA is the most commonly used serological marker for the diagnosis of prostate cancer. The United States Food and Drug Administration (FDA) has approved PSA detection as an indicator for general survey of men over 50 years of age. The PSA value of normal men is <4 ng/ml. The results of the present meta-analysis indicated that the expression of caveolin-1 in prostate cancer with PSA >10 ng/ml was 2.09 times higher than that with PSA ≤10 ng/ml, suggesting that the expression of caveolin-1 increases gradually with carcinogenesis, and is a promotive factor for the occurrence and development of prostate cancer.

Univariate analysis showed that caveolin-1 overexpression in prostate cancer patients was significantly correlated with low grade differentiation (OR=2.74, p<0.01), advanced clinical stage (OR=2.77, p<0.01), and positive lymph node metastasis (OR=3.91, p<0.01). Caveolin-1 expression is known to be closely related to the invasion and metastasis of prostate cancer. Caveolin-1 overexpression also predicts poor survival in patients with prostate cancer. The patients with high expression of caveolin-1 had a shorter survival time than those with normal expression of caveolin-1, which can be used as an important reference index to predict the prognosis in prostate cancer.

Tahir SA et al. (31) reported that elevated preoperative levels of serum Caveolin-1 predicted decreased time to cancer recurrence. And Steiner I et al. (34) also reported that lower Caveolin-1 mRNA expression was associated with biochemical recurrence. However, Mathieu R et al. (17) indicated that Caveolin-1 was an independent predictor of BCR in multivariable analyses that adjusted for the effects of standard clinicopathological features, but, it did not add prognostically relevant information to established predictors of BCR, limiting its use in clinical practice. Although the above results indicate that Cav-1 is closely related to prostate cancer recurrence, the application of Caveolin-1 in prostate cancer recurrence still needs further research to verify.

This is the first study to report the different expression levels of caveolin-1 in prostate cancer tissues, precancerous lesion tissues (HGPIN) and normal prostate tissues, confirming that caveolin-1 is overexpressed in prostate cancer and HGPIN tissues. In prostate cancer patients, the expression of caveolin-1 was closely related to cancer cell differentiation, clinical stage, lymph node metastasis, and serum PSA level. Similar to the results of Liu et al. (35), this study also found that overexpression of caveolin-1 predicts poor prognosis in prostate cancer patients after radical prostatectomy.

This study had a few limitations. First, only published English and Chinese studies were enrolled in the meta-analysis, which may have led to publication bias. Second, due to limitation of included studies, the sample size enrolled in the meta-analysis was relatively small. Third, due to different detection methods and criteria, the enrolled studies had some heterogeneity. Fourth, because of different race and age, the subjects included in each study were also a source of heterogeneity. Fifth, because the included articles did not report detailed survival data, we could only use the Kaplan-Meier curve in the survival analysis to infer the corresponding results, which may have overestimated or underestimated the real survival data. Hence, the results of this meta-analysis should be verified by additional larger sample size and well-designed studies.

## Conclusion

The results of this meta-analysis demonstrated that caveolin-1 is overexpressed in prostate cancer. The overexpression of caveolin-1 is associated with an unfavorable clinicopathological status, including a high level of serum PSA, low degree of differentiation, advanced clinical stage, and positive lymph node metastasis. Hence, the overexpression of caveolin-1 predicts poor survival. Caveolin-1 could be a new candidate tumor marker for prostate cancer, which could provide clinical basis for the diagnosis and prognosis evaluation.

## Data Availability Statement

All datasets presented in this study are included in the article/supplementary material.

## Author Contributions

All authors listed have made a substantial, direct, and intellectual contribution to the work and approved it for publication.

## Conflict of Interest

The authors declare that the research was conducted in the absence of any commercial or financial relationships that could be construed as a potential conflict of interest.
